# A Low-Computational-Complexity Digital Predistortion Model for Wideband Power Amplifier

**DOI:** 10.3390/s24216941

**Published:** 2024-10-29

**Authors:** Xu Lu, Qiang Zhou, Lei Zhu, Zhihu Wei, Yaqi Wu, Zunyan Liu, Zhang Chen

**Affiliations:** 1 School of Electronics and Information Engineering, Nanjing University of Information Science and Technology, Nanjing 210007, China; 202212490452@nuist.edu.cn (X.L.); 202212490446@nuist.edu.cn (Y.W.); 2 The Sixty-Third Research Institute, National University of Defense Technology, Nanjing 210007, China; zhouqiang63@nudt.edu.cn (Q.Z.); zhuleidz@163.com (L.Z.); plawzh@163.com (Z.W.);

**Keywords:** digital predistortion, wideband power amplifier, composition piecewise memory polynomial, complexity, linearization

## Abstract

This paper proposes a Composition Piecewise Memory Polynomial (CPMP) digital predistortion model based on a Vector Switched (VS) behavioral model to address the challenges of severe nonlinearity and strong memory effects in wideband power amplifiers (PAs). To tackle this issue, two thresholds are calculated and used to segment the envelope values of the input signal according to the nonlinear distortion characteristics of the PA. In this approach, a Generalized Memory Polynomial (GMP) model is employed for the lower segment, a Memory Polynomial (MP) model is employed for the middle segment, and a higher-order GMP model is employed for the upper segment. By sharing the fundamental MP among the proposed segmented models and leveraging a design methodology that configures different cross terms, memory depths, and polynomial orders for each segment, this model achieves superior linearization performance while simultaneously reducing the computational complexity associated with model extraction. The experimental results demonstrate that the adjacent channel power ratio (ACPR) of the predistorted PA output signal using the proposed model improves from −36 dBc to −54 dBc, matching the performance of the GMP model. Furthermore, this performance is 0.5 dBc better than the Piecewise Dynamic Deviation Reduction (PDDR) and Decomposed Vector Rotation (DVR) models. Notably, the complexity of the proposed parameter extraction process is 28.8% of the DVR model, 21.79% of the GMP model, and 12.83% of the PDDR model.

## 1. Introduction

In wireless communication systems, it is difficult for the transmitter to maintain balance with the linearization and efficiency of the power amplifier. When high-order modulated signals with a higher bandwidth are used, the system places higher demands on the linearity performance of the transmitter. To address this problem, the digital predistortion (DPD) technique is widely used in wireless communication systems due to its high linearization capability and flexibility in application [1,2].

The nonlinear behavior model serves as the cornerstone of digital predistortion techniques [3,4,5,6], with numerous memory nonlinear models derived from the Volterra series having been proposed. Among these, the Memory Polynomial (MP) model features a straightforward structure that effectively compensates for nonlinear distortion [7,8]; however, its linearization performance in wideband and high peak-to-average power ratio (PAPR) input signal scenarios is somewhat lacking. Conversely, the Generalized Memory Polynomial (GMP) model addresses this limitation by incorporating additional cross terms with varying memory depths, enabling it to provide effective predistortion correction for wideband and high PAPR signals [9]. The Vector Switched (VS) model has been introduced to enhance the efficiency of power amplifier architectures [10], but the number of parameters required escalates exponentially with the number of segments used. A novel technique for identifying the parameters of a digital predistorter, termed Iterative Learning Control Digital Predistortion (ILC-DPD), has been developed by J. Chani-Cahuana, demonstrating better linearization performance compared to indirect learning architecture (ILA) and direct learning architecture (DLA) [11]. Additionally, C. Kantana has proposed an auto-tuning approach designed to optimize the performance of dual-input Doherty power amplifiers, maximizing power efficiency while adhering to linearity specifications [12]. Me ngozzi M. introduced an automatic parameter-setting method utilizing coordinate descent or Bayesian optimization to enhance power addition efficiency while maintaining appropriate linearity for wideband modulated signals [13]. For more complex linearization scenarios, such as envelope tracking (ET) systems where the amplifier drain voltage fluctuates dynamically with the input signal’s envelope, the inherent nonlinear characteristics are intensified even as efficiency is improved [14]. In [15], a Piecewise Dynamic Deviation Reduction (PDDR) model based on the Volterra series has shown promising performance when applied to ET systems. Furthermore, G. P. Gibiino has proposed a real-time inversion algorithm for the two-input behavioral model of supply modulated RF power amplifiers, enabling accurate linearization of the envelope tracking power amplifier in real-time settings [16].

In summary, the Digital Predistortion (DPD) model often requires the introduction of additional parameters to accurately capture the complex behavior of power amplifiers, leading to increased complexity and computational overhead. This escalation poses challenges and costs for engineering implementations, thereby limiting the widespread application of predistortion techniques. To tackle this issue, this paper proposes a Composition Piecewise Memory Polynomial (CPMP) model based on the Vector Switched (VS) model.

In this approach, two thresholds are established according to the nonlinear distortion characteristics of the amplifier, and segmentation is performed based on the envelope of the input signal. Specifically, the Generalized Memory Polynomial (GMP) model is employed for the lower segment, the Memory Polynomial (MP) model is employed for the middle segment, and a higher-order GMP model is employed for the upper segment. These segments are ultimately recombined to produce a predistorted output. The CPMP model not only provides a more accurate representation of the nonlinear characteristics in wideband scenarios but also simplifies the parameter extraction process. Additionally, it mitigates the issue of discontinuities around segmentation points that are commonly encountered in conventional segmentation models.

The structure of the remainder of this paper is organized as follows: Section 2 discusses the characteristics of the MP, GMP, and segmentation models. Section 3 presents the structure of the proposed CPMP model, detailing the thresholding method, parameter extraction process, and complexity analysis. Section 4 analyzes and simulates the linearization performance of the proposed model under varying thresholds. In Section 5, the actual power amplifier is utilized to validate the linearization performance of the proposed model in comparison to other models, along with an analysis of the computational complexity of parameter extraction for the different models. Finally, Section 6 presents the conclusions drawn from this study.

## 2. Nonlinear Array System Model

The MP model is used to characterize the nonlinear behavior of the PA by removing the cross terms with inconsistent memory depths of the Volterra stages, and this can be written as Formula (1).

(1) $$y_{mp}=\sum_{k=1}^{K}\sum_{q=0}^{Q-1}a_{kq}x(n-q){\left|x(n-q)\right|}^{k-1}$$

where *K* represents the nonlinear order, *Q* represents the memory depth, *a_kq_* denotes the model parameters, and |•| is a complex number taking an absolute value operation.

The MP model has the advantages of being flexible and simple to implement for modeling PAs, but the performance degrades when targeting the nonlinear distortion due to strong memory characteristics [17].

The GMP model can implement predistortion for wideband signals. This model increases the cross terms of leading and lagging compared to MP, which improves the linearization performance and increases the parameter extraction complexity of the model, which can be written as Formula (2).

(2) $$\begin{array}{cc} y_{gmp} & =\sum_{k=1}^{K}\sum_{q=0}^{Q-1}a_{kq}x(n-q){\left|x(n-q)\right|}^{k-1}\\ & +\sum_{k=1}^{K_{b}}\sum_{l}^{L_{b}}\sum_{q=1}^{Q_{b}}b_{klq}x(n-l){\left|x(n-l-q)\right|}^{k-1}\\ & +\sum_{k=1}^{Kc}\sum_{l}^{L_{c}}\sum_{q=1}^{Q_{c}}c_{klq}x(n-l){\left|x(n-l+q)\right|}^{k-1}\;v \end{array}$$

where *K*, *K_b_*, and *Kc* represent the nonlinear order, *Q*, *Q_b_*, and *Qc* represent the memory depth, *a_kq_*, *b_klq_*, and *c_klq_* are the model parameters, |•| is the complex number taking absolute value operation, *L_b_* is the lagging depth, and *Lc* is the leading depth.

The GMP model allows for the modeling of the strong memory PAs’ behavior caused by wideband signals. However, the complexity of this model increases considerably when applied directly to ET systems, which have different distortion characteristics at different input power levels.

**Figure 1:**
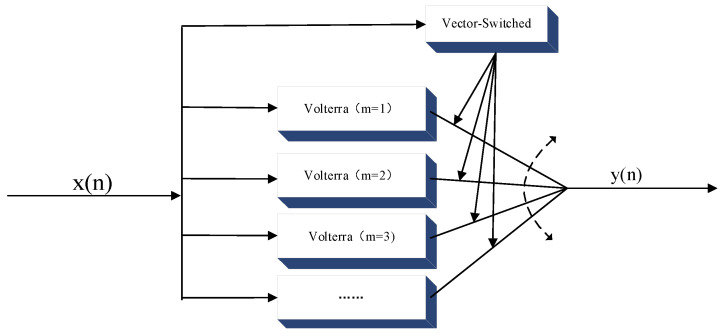
VS model structure diagram.

As shown in Figure 1, for an example of a VS model divided into multiple intervals, the input interval is divided into regions, where each region uses a separate model to calculate the output, and each region has separate parameters that are identified according to different parts of the input/output space.

The method based on VS can simplify the nonlinear modeling of wideband signals with a strong memory effect, and the basic idea of the method is to segment the nonlinear distortion curves according to the power magnitude of the input signals and use different functions to fit each segment of the curve, achieving the modeling of complex nonlinear characteristics, as shown in Formula (3).

(3) $$y=\left\{\begin{array}{ll} F_{1}(•) & \left|x(n)\right|\leq \lambda_{1}\\ F_{2}(•) & \lambda_{1}<\left|x(n)\right|\leq \lambda_{2}\\ \;\;\vdots & \;\;\vdots \\ F_{M}(•) & \left|x(n)\right|>\lambda_{M} \end{array}\right.$$

where *x*(*n*) is the complex form of the input I/Q signal, |•| is the absolute value taken on the complex number, i.e., the amplitude of the input signal, *F_M_*(•) is the nonlinear fitting function for the *M*th interval, *M* is the number of segments, *λ* is the threshold of the segments, the commonly used *F*(•) denotes the polynomial, MP, and DDR models, etc. [8,15,18,19].

From Formula (3), the composition of the segmentation function directly affects the performance of the linearization, so the selection of each segmentation function becomes very important. In addition, the complexity of the model is positively related to the number of segments, and the number of segments must be chosen reasonably [15,20].

**Figure 2:**
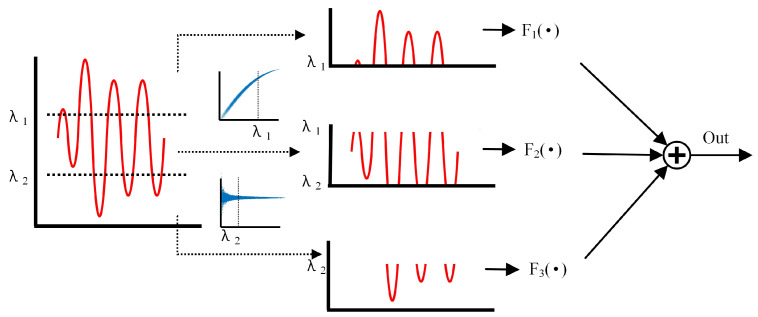
Signal decomposition and recombination.

Therefore, according to the amplitude distortion characteristics and phase distortion characteristics caused by the PA, we divide the input signal into three segments according to the power level of the input signal, and its segmented curve fitting is shown in Figure 2. On this basis, different fitting functions are selected according to the power of the input signal, and the model of this paper is proposed.

## 3. Composition Piecewise Memory Polynomial Model

### 3.1. Model Structure

The amplitude distortion and phase distortion due to the nonlinear behavior of the PA are shown in Figure 1. When the power of the input signal is small, the output signal phase error of the PA is more easily dispersed by the influence of memory. When the PA is driven by a high-power signal, the gain compression is significant and becomes the main factor of the nonlinear distortion. According to the above characteristics, we divide the nonlinear distortion into three parts with different functions, and the thresholds of the segments are shown in Formula (4).

(4) $$\Omega =\left\{\lambda_{1},\;\;\;\;\lambda_{2}\right\}$$

where *λ* is the amplitude discrimination threshold of the input signal, λ_1_ < λ_2_. In Section 3.3, we will discuss the different effects of different threshold selection on the output signal of the PA.

The MP model is simple and easy to implement, and the GMP model can compensate for complex memory distortion and amplitude distortion. Therefore, the MP model can be combined with different cross terms to form GMP1 and GMP2 in different distortion cases.

The proposed model consists of three parts, as shown in Formula (5).

(5) $$y=\left\{\begin{array}{ll} F_{gmp1}(•) & \left|x(n)\right|\leq \lambda_{1}\\ F_{mp}\;\;(•) & \lambda_{1}<\left|x(n)\right|\leq \lambda_{2}\\ F_{gmp2}(•) & \left|x(n)\right|>\lambda_{2} \end{array}\right.$$

where *x*(*n*) is the complex form of the input I/Q signal, |•| is the absolute value that has taken over the complex number, *F_gmp_*_1_(•) is the nonlinear fitting function for the first interval, *F_mp_*(•) is the nonlinear fitting function for the second interval, *F_gmp_*_2_(•) is the nonlinear fitting function for the third interval, and *λ* is the threshold for segmentation.

Based on the above discussion, when the power of the input signal is small, the phase distortion of the signal is more dispersive, so the GMP model containing the complex cross terms is chosen as the predistortion function for the first part.

When the power of the input signal is moderate, the MP model with relatively low complexity is chosen to compensate for the nonlinear characteristics of this part, as shown in Formula (1). The operator matrix formed by MP is shown in Formula (6).

(6) $$U_{mp}=\left[\begin{array}{ccc} x(n) & \ldots & x(n-q){\left|\;x(n-q)\right|}^{k-1}\\ \vdots & \ddots & \vdots \\ x(1)\; & \cdots & 0 \end{array}\right]$$

When the power of the input signal is increasing, the output signal of the PA starts to compress significantly, and the nonlinear distortion is severe. Therefore, the high-order GMP model can be selected in this part, as shown in Formula (2).

The operator matrix formed by GMP is shown in Formula (7).

(7) $$U_{gmp}=\left[U_{mp}\left|\begin{array}{ccc} x(n-1)\left|\;x(n-2)\right| & \ldots & x(n-l){\left|\;x(n-l+q)\right|}^{k-1}\\ \vdots & \ddots & \vdots \\ 0 & \cdots & 0 \end{array}\right.\right]$$

**Figure 3:**
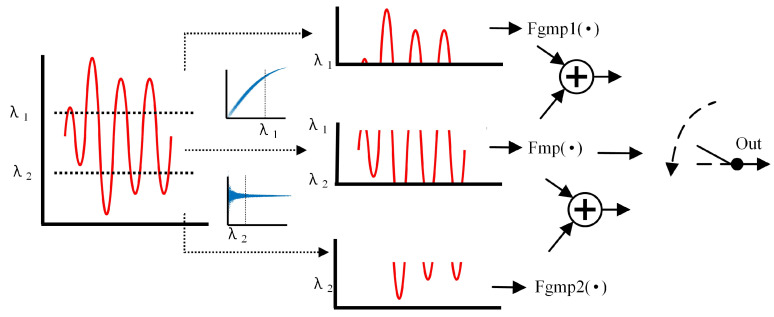
The structure of CPMP.

The basic function we proposed is MP; in other words, GMP models reuse the coefficients of the part of MP, and its structure among the three functions is shown in Figure 3.

In summary, we predistort the input signal into segments according to the level of power. According to the basic structure of Figure 3 and the structure of the segmentation function shown in Figure 2, the original input signal is first judged by its power level, and then a different model is entered and the output signal is synthesized according to the division of the power threshold, as shown in Formula (8).

(8) $$y=F_{mp}\;(x(n))+\left\{\begin{array}{ll} F_{gmp1}(x(n)) & \left|x(n)\right|\leq \lambda_{1}\\ 0\; & \lambda_{1}<\left|x(n)\right|\leq \lambda_{2}\\ F_{gmp2}(x(n)) & \left|x(n)\right|>\lambda_{2} \end{array}\right.$$

where *F_mp_* is the common MP function, *F_gmp_*_1_ is the cross term of the GMP1 model, and *F_gmp_*_2_ is the cross term of the GMP2 model.

### 3.2. Model Parameter Extraction

**Figure 4:**
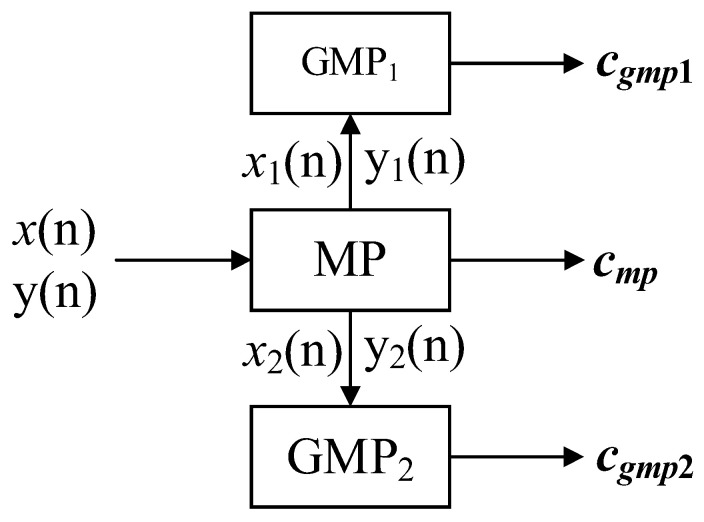
The CPMP model parameter extraction process.

The model parameter extraction process is shown in Figure 4; the number extraction process is divided into three steps.

#### 3.2.1. MP Model Coefficient Solution

A generalized indirect learning architecture can be used to extract paraments. Then, we copy parameters *c_mp_* into the predistorter.

The general indirect learning architecture can be adopted. The input signal x of the power amplifier is used as the desired signal to obtain the back-inversion of the power amplifier, and the solved parameter *c_mp_* is copied into the predistorter. The MP model parameter extraction process is shown in Formula (9).

(9) $$U_{mp}\cdot c_{mp}=X$$

where *U_mp_* consists of the output signal *y* and *c_mp_* denotes the paraments of the MP model.

#### 3.2.2. GMP Model Coefficient Solution

We extract the coefficients composed of GMP cross terms according to the piecewise threshold. Let *U_gmp_* be the matrix of operators composed of the cross terms of the GMP model and let the parameter of the searched MP also be used as the parameter of the first term of the GMP, as shown in Formula (10).

(10) $$\left\{\begin{array}{l} U_{mp}\cdot c_{mp}+U_{gmp1}\cdot c_{gmp1}=X_{1}\\ U_{mp}\cdot c_{mp}+U_{gmp2}\cdot c_{gmp2}=X_{2} \end{array}\right.$$

where *U_gmp_*_1_/*U_gmp_*_2_ are the values of the output signal *y* in given intervals, *X*_1_/*X*_2_ are the desired signals corresponding to the output signal y in given intervals, and *c_mp_* is the parameter of the MP model. *c_gmp_* is the parameter of the cross term of the GMP model, as shown in Formula (11).

(11) $$U_{gmp}\cdot c_{gmp}=X-U_{mp}\cdot c_{mp}$$

The model parameters are determined by the method of least squares (LS), as shown in Formula (12).

(12) $$c={\left(U^{H}U\right)}^{-1}U^{H}X$$

where *U* is the operator matrix constructed by the model, (•)^H^ is the conjugate transpose operation of the matrix, and (•)^−1^ is the inverse operation of the matrix.

From the above discussion, the core of the proposed model is MP, which can better compensate for the nonlinearity of the PA in the middle section, so the ideal result of the MP model in the middle section is shown in Formula (9), and at this time Formula (13) can be obtained by substituting the segmentation point in Formula (10).

(13) $$U_{gmp}\cdot c_{gmp}=X-U_{mp}\cdot c_{mp}\approx 0$$

Therefore, the effect of the discontinuity near the segmentation point is directly related to the selection of the segments, and the effect of the discontinuity near the segmentation point can be reduced by selecting a suitable segmentation point.

### 3.3. Parameter Extraction Complexity Comparison

In order to analyze the computational complexity of the CPMP model parameter extraction process, in the process of parameter extraction, the complexity of complex multiplication is much larger than that of complex addition, so the complexity of addition is ignored [18]. Assuming that there are two thresholds, *λ*_1_ and *λ*_2_ (*λ*_1_ < *λ*_2_), the signal is divided into three sections. Firstly, the sampling signals in the three sections share the MP model for parameter extraction, and then the sampling points in the interval with amplitude less than *λ*_1_ and the interval with amplitude greater than *λ*_2_ use the cross term of the GMP model for new parameter extraction, and the number of model parameters is the same. The number of sample points is *N*, the number of parameters of the GMP model is *L*, the number of parameters of the MP term in the CPMP model is *L*/2, the number of parameters of the GMP cross term is *L*/2, and the number of sample points of the cross term is *N*_1_ and *N*_2_, respectively. The LS algorithm shown in Formula (12) is mainly divided into four parts. *S*_1_ is matrix multiplication, *S*_2_ is matrix inversion, *S*_3_ is matrix multiplication, and *S*_4_ is matrix multiplication.

As shown in Table 1 and Table 2, the LS algorithm is divided into matrix operations, and the number of complex multiplication operations of GMP and CPMP is compared. Since *N*_1_ and *N*_2_ are the sampling points on the cross terms of the CPMP model, the sum of *N*_1_ and *N*_2_ is less than *N*, so the multiplication calculation amount of each individual matrix in Table 2 is smaller than that of the matrix in Table 1, so the multiplication calculation amount required for parameter extraction of the CPMP model is smaller than that of the GMP model.

**Figure 5:**
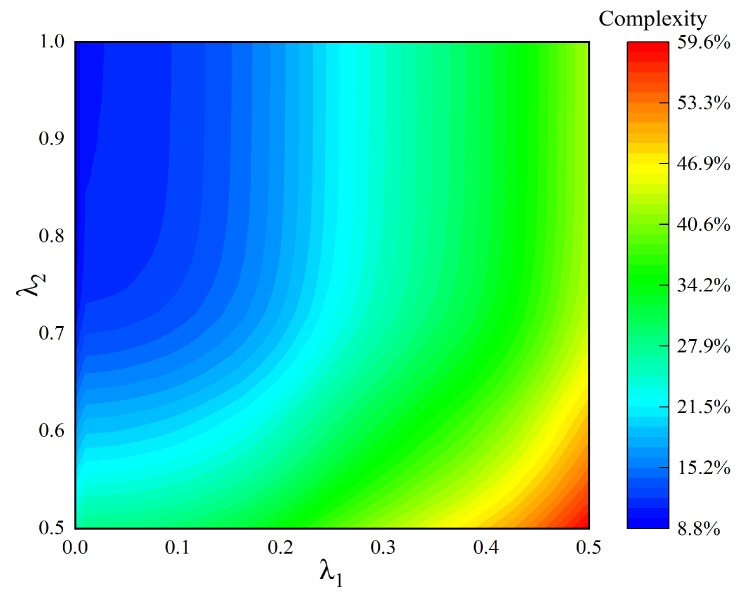
Extraction complexity of different threshold parameters.

As shown in Figure 5, the computational complexity of the parameter extraction of the CPMP model relative to the GMP model under different thresholds is shown. When *λ*_1_ decreases and *λ*_2_ increases, the number of sampling points using the GMP model to extract parameters gradually decreases, and the complexity of parameter extraction gradually decreases. Similarly, when *λ*_1_ increases and *λ*_2_ decreases, the complexity of parameter extraction gradually increases. Since the proposed CPMP model is based on the MP model, the GMP model reuses part of the MP coefficients. Therefore, the minimum complexity is to use the MP model parameter extraction operation for all sampled signals, that is, there is no GMP model for parameter extraction; the highest complexity is the sum of the parameters extracted by the cross term of the GMP model after the MP model is used to extract the parameters of all the sampled signals.

## 4. Simulation Results

In order to analyze the performance of the proposed model under different thresholds, simulation experiments were carried out. Among them, the power amplifier model is the Winner–Saleh model, the input signal is a 16 QAM signal with a bandwidth of 50 MHz, and the PAPR is 6.9 dB; the proposed model’s structure is shown in Table 3.

**Figure 6:**
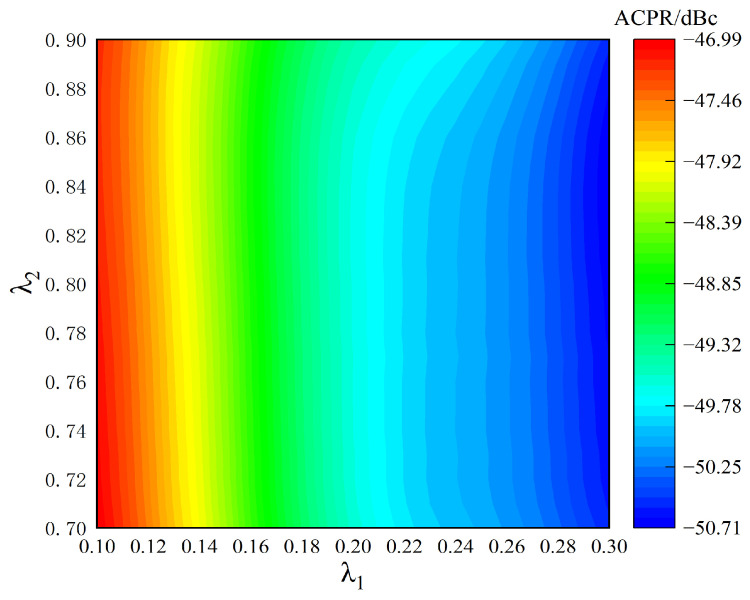
Different threshold power amplifier output signal ACPR value.

As shown in Figure 6, the adjacent channel power ratio (ACPR) of the corresponding power amplifier output signal under different thresholds is compared. Due to the uneven amplitude distribution of the sampled signal, the number of sampling points in the range of amplitude less than *λ*_1_ is relatively large, which affects the performance of parameter extraction and then affects the improvement effect of ACPR. Therefore, when *λ*_1_ increases and *λ*_2_ remains unchanged, the complexity of the parameter extraction increases, and the improvement effect of ACPR becomes better. However, the number of sampling points in the interval with amplitude greater than *λ*_2_ is relatively small, which has little effect on the improvement effect of ACPR. When *λ*_1_ remains unchanged and *λ*_2_ increases, the improvement effect of ACPR has no obvious effect.

**Figure 7:**
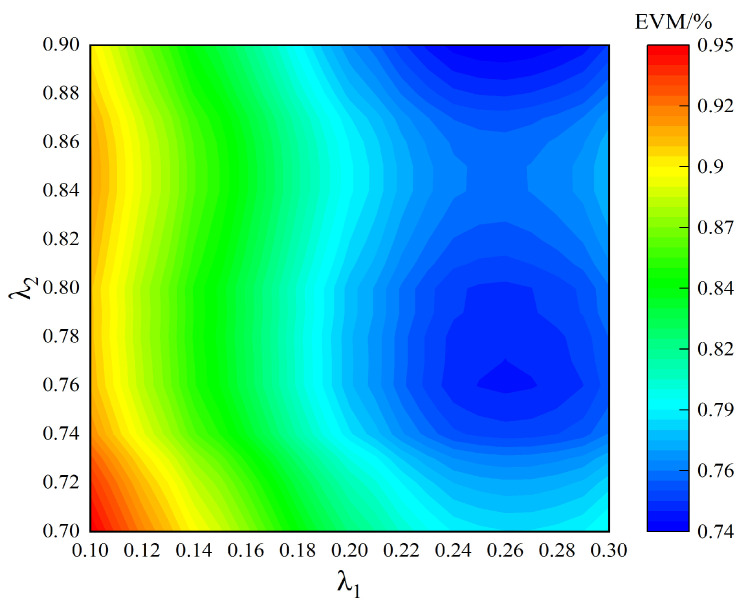
Different threshold power amplifier output signal EVM value.

As shown in Figure 7, the error vector magnitude (EVM) of the corresponding power amplifier output signal under different thresholds is compared. There is no significant difference in the EVM between different thresholds, which meets the actual application requirements.

According to the comparison of the output signal performance corresponding to different thresholds in Figure 5, Figure 6 and Figure 7, in the case of the same threshold, it is impossible to ensure that all three can achieve an optimal performance. Therefore, the threshold selected for the actual power amplifier test should be a good compromise between the three.

## 5. Experimental Results

**Figure 8:**
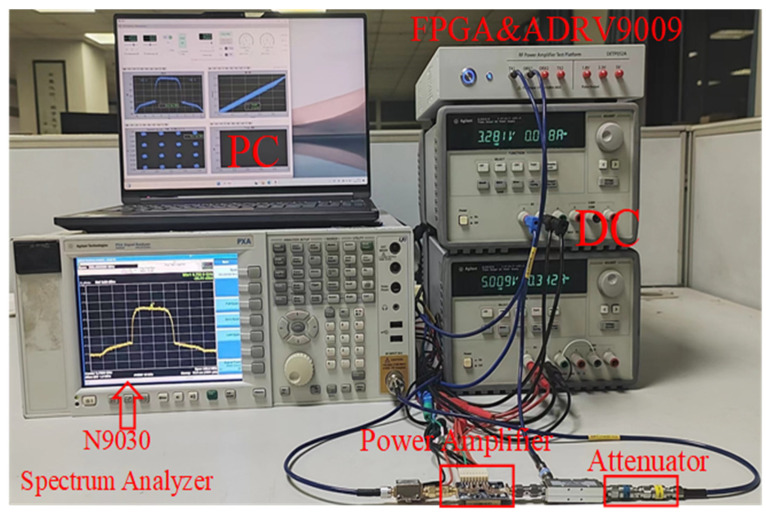
Measurement setup for the DPD scheme.

In order to verify the performance of the CPMP model proposed in this paper, we carried out experimental verification. The experimental test uses a digital radio frequency experimental platform, as shown in Figure 8. The baseband signal is generated by the PC side and downloaded to the FPGA. After up-conversion by ADI’s RF sub-board ADRV9009, it is input to the power amplifier. The output signal of the power amplifier returns to the digital radio frequency experimental platform through the attenuator, showing the spectrum and constellation diagram of the signal.

In this experiment, the main power amplifier is 5004L from SKYWORKS company. The transistor adopts the GaN process, and its output power is 30 dBm. The measured signal is a standard 5G New Ratio (NR) signal, and the baseband signal modulation mode is 16QAM. The signal bandwidth is 50 MHz, the carrier frequency is 5.7 GHz, the PAPR is 8.5 dB, and the number of sampled signals *N* is 40,000. According to the results of the simulation experiments, in order to achieve a compromise effect on the performance of the output signal of the power amplifier, the CPMP model is constructed as shown in Table 4.

The normalized mean square error (NMSE) of the power amplifier output signal and the power amplifier input signal after predistortion is used to evaluate the fitting ability of different digital predistortion models to the power amplifier. As shown in Table 5, the coefficients of different models are compared with NMSE under the condition that the same nonlinear order K and memory depth M are adopted. The nonlinear order and memory depth of the GMP model and CPMP model are shown in Table 4. Among them, there are 90 parameters in the PDDR model, 60 parameters in the DVR model, 69 parameters in the GMP model, 20 basic items in the CPMP model, and 49 cross items in the GMP1 and GMP2. The normalized mean square error (NMSE) of the different models is similar, and there is no obvious difference. The CPMP model has 21 fewer coefficients than the PDDR model and 9 more than the DVR model.

**Figure 9:**
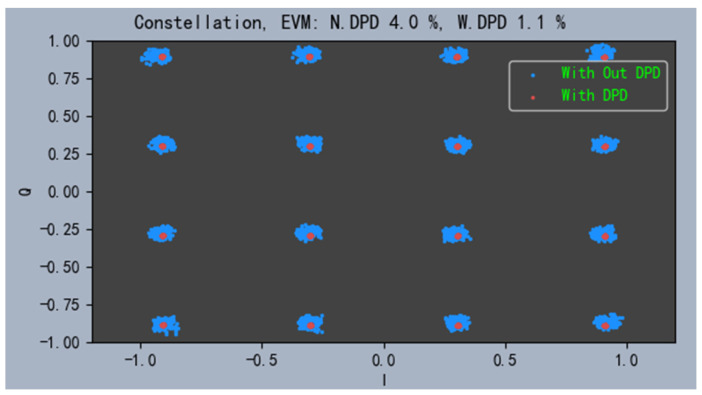
Constellation.

The constellation diagram of the power amplifier output signal using the CPMP model is shown in Figure 9. The EVM of the CPMP model is reduced from 4% to 1.1%, which has a significant improvement.

**Figure 10:**
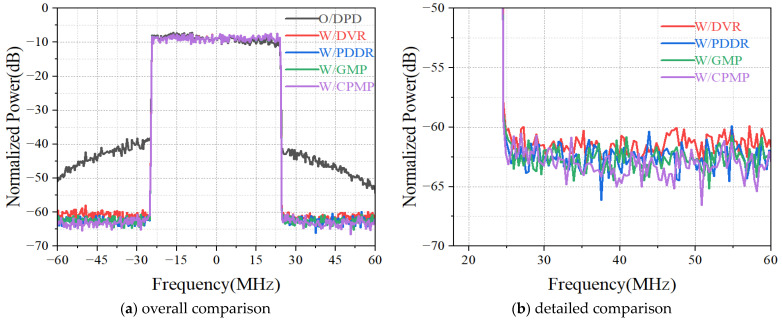
The spectral comparison of the amplifier output signals before and after predistortion.

Figure 10 shows the spectral comparison of the amplifier output signals before and after predistortion with different models.

The corresponding ACPR and EVM are given in Table 6. The experiment results show that the ACPR of the CPMP model is improved from −36 dBc to −54 dBc, which is 0.5 dBc higher than the PDDR and DVR model, which is comparable to that of the GMP model. Also, there is no significant difference in the EVM.

Table 7 compares the number of multiplication operations required for different model parameter extraction and the complexity of the different model relative to the PPDR model. The complexity of the CPMP model is 28.8% of the DVR model, 21.79% of the GMP model, and 12.83% of the PDDR model, respectively. In the case of comparable linearization performance, the computational complexity of parameter extraction is significantly reduced.

In summary, under the same conditions, compared with other models, the CPMP model proposed in this paper has considerable power amplifier fitting ability and linearity improvement ability, but the number of multiplication operations required for parameter extraction is far lower than that of other models, saving hardware implementation costs and effectively suppressing in-band and out-of-band distortion.

## 6. Conclusions

Aiming at solving the problem of serious nonlinearity and strong memory effect correction in a broadband power amplifier, this paper proposes a digital predistortion technology based on a composite piecewise memory polynomial model based on a vector selection model. Firstly, the structure and characteristics of the MP, GMP, and VS models are introduced. Then, a composite piecewise memory polynomial model is proposed. The proposed model can accurately model the nonlinear characteristics of power amplifiers while ensuring low parameter extraction complexity. In addition, the influence of discontinuity near the segmentation point is reduced. Finally, the simulation test is carried out to analyze the linearization performance of the output signal of the power amplifier under different segmentation thresholds of the proposed model. Through the actual power amplifier experiment, the linearization performance of the proposed model is compared with other models, and the parameter extraction complexity of different models is compared. The experimental results show that the linearization performance of the proposed model is comparable to that of the GMP model and better than that of the DVR and PDDR models. The parameter extraction complexity of the CPMP model is 28.8% of the DVR model, 21.79% of the GMP model, and 12.83% of the PDDR model, respectively. Therefore, the proposed model can dynamically adjust the threshold for wideband power amplifiers to achieve a better linearization effect, reduce the complexity of the parameter extraction process, and have stronger robustness.

## Figures and Tables

**Table 1:** Complex multiplications for each matrix operation of GMP.

Matrix Operation	Multiplications
*S*_1_ = *U^H^U*	*N* × *L* × *L*
*S*_2_ = (*S*_1_)^−1^	*L* × *L* × *L*
*S*_3_ = *S*_2_*U^H^*	*N* × *L* × *L*
*S*_4_ = *S*_3_*X*	*N* × *L*
*GMP*	2 × *N* × *L*^2^ *+ N* × *L* + *L*^3^

**Table 2:** Complex multiplications for each matrix operation of CPMP.

Matrix Operation	Multiplications
*S*_1_ = *U^H^U*	1/4 × *L*^2^ × (*N* + *N*_1_ + *N*_2_)
*S*_2_ = (*S*_1_)^−1^	3/8 × *L* × *L* × *L*
*S*_3_ = *S*_2_*U^H^*	1/4 × *L*^2^ × (*N* + *N*_1_ *+ N*_2_)
*S*_4_ = *S*_3_*X*	1/2 × *L* × (*N* + *N*_1_ *+ N*_2_)
*CPMP*	1/2 × (*L*^2^ + *L*)(*N* + *N*_1_ + *N*_2_) + 3/8 × *L*^3^

**Table 3:** The selection of different thresholds of the proposed model.

λ_1_ = 0.1~0.3	λ_2_ = 0.7~0.9
*F*_mp_	*K* = 5, *Q* = 3
*F*_gmp1_	*K_b_* = 7, *Q_b_* = 3, *L_b_* = 1
*F*_gmp2_	*Kc* = 7, *Qc* = 3, *Lc* = 1

**Table 4:** The structure of the proposed model.

λ_1_ = 0.25	λ_2_ = 0.8
*F*_mp_	*K* = 5, *Q* = 3
*F*_gmp1_	*K*_b_ = 7, *Q*_b_ = 3, *L*_b_ = 1
*F*_gmp2_	*Kc* = 7, *Qc* = 3, *Lc* = 1

**Table 5:** Comparison of NMSE and number of coefficients between different models.

Model	K = 5, Q = 3	
	No. Coefficients	NMSE (dB)
PDDR	90	−34.71
DVR	60	−34.69
GMP	69	−34.73
CPMP	69	−34.71

**Table 6:** ACPR and EVM.

Model	Lower ACPR/dB	Upper ACPR/dB	EVM/%
O/DPD	−34.9	−37.9	4.0
PDDR	−53.8	−53.9	1.3
GMP	−54.5	−54.6	1.1
DVR	−53.9	−53.8	1.2
CPMP	−54.5	−54.2	1.1

**Table 7:** The complexity of model parameter extraction.

Model	Multiplications	Complexity/%
PDDR	652,329,000	100
GMP	383,968,509	58.86
DVR	290,616,000	44.55
CPMP	83,705,000	12.83

## Data Availability

Data are contained within the article.
